# The decline in youth drinking in England—is everyone drinking less? A quantile regression analysis

**DOI:** 10.1111/add.14824

**Published:** 2019-12-01

**Authors:** Melissa Oldham, Sarah Callinan, Victoria Whitaker, Hannah Fairbrother, Penny Curtis, Petra Meier, Michael Livingston, John Holmes

**Affiliations:** ^1^ School of Health and Related Research University of Sheffield Sheffield UK; ^2^ Centre for Alcohol Policy Research LaTrobe University Melbourne Australia; ^3^ School of Nursing and Midwifery University of Sheffield Sheffield UK

**Keywords:** Alcohol consumption, collectivity, polarization, trend analysis, young people, youth drinking

## Abstract

**Background and Aims:**

Youth alcohol consumption has declined significantly during the past 15 years in many high‐income countries, which may have significant public health benefits. However, if the reductions in drinking occur mainly among lighter drinkers who are at lower risk, then rates of alcohol‐related harm among young people today and adults in future may not fall in line with consumption. There is conflicting evidence from Swedish school studies, with some suggesting that all young people are drinking less, while others suggest that alcohol consumption among heavier drinkers may be stable or rising while average consumption declines. This paper extends the geographical focus of previous research and examines whether the decline in youth drinking is consistent across the consumption distribution in England.

**Design:**

Quantile regression of 15 waves of repeat cross‐sectional survey data.

**Setting:**

England, 2001–16.

**Participants:**

A total of 31 882 schoolchildren (50.7% male) aged 11–15 who responded to the Smoking Drinking and Drug Use among Young People surveys.

**Measurements:**

Past‐week alcohol consumption in UK units at each fifth percentile of the consumption distribution.

**Findings:**

Reductions in alcohol consumption occurred at all percentiles of the consumption distribution analysed between 2001 and 2016, but the magnitude of the decline differed across percentiles. The decline in consumption at the 90th percentile [β = −0.21, confidence interval (CI) = −0.24, −0.18] was significantly larger than among either lighter drinkers at the 50th percentile (β = −0.02, CI = −0.02, −0.01) or heavier drinkers at the 95th percentile (β = −0.16, CI = −0.18, −0.13).

**Conclusions:**

Alcohol consumption among young people in England appears to be declining across the consumption distribution, and peaks among heavy drinkers. The magnitude of this decline differs significantly between percentiles of the consumption distribution, with consumption falling proportionally less among the lightest, moderate and very heaviest youth drinkers.

## Introduction

Youth alcohol consumption has fallen sharply in most high‐income countries throughout Europe 1, 2, 3, 4, 5, 6, North America 1 and Australasia 7, 8. The analyses in this paper focus on England, where the proportion of 8–12‐year‐olds who have ever had an alcoholic drink fell from 25% in 2002 to 4% in 2016, while a separate survey shows a concurrent fall among 11–15‐year‐olds from 61% in 2003 to 38% in 2014 5. Those young people in England who do drink are starting to do so at a later age and are consuming alcohol less often and in smaller quantities 5. Data from other countries where youth drinking is in decline present a largely similar picture 2.

Adolescent drinking is associated with a range of negative health outcomes, including brain damage and neurocognitive deficits, which can affect intellectual development 9. Similarly, the likelihood of developing alcohol use disorders later in life increases with younger ages of alcohol initiation 9, 10. Youth drinking is also linked to short‐term harms, such as accidents 10, 11 and risky behaviours, which could lead to problems including sexually transmitted infections, injuries and criminality or victimization 11, 12, 13, 14, 15. As such, declines in youth alcohol consumption should lead to significant improvements in public health. However, these potential benefits may be lessened if the declines in drinking are not distributed evenly throughout the population or are concentrated among those at lowest risk of harm.

The potential for youth drinking trends to polarize, with reduced consumption among lighter drinkers and stable or even increased consumption among heavier drinkers, is contrary to Skog's influential theory of the collectivity of drinking cultures. This theory states that, through social diffusion processes, changes in per‐capita alcohol consumption tend to result from individuals changing their consumption in concert across the population 16. Until recent years, robust empirical validations of Skog's theories were lacking; however, in 2014, Rossow *et al*. demonstrated the apparent collectivity of adult consumption trends in several countries 17. Since then, several studies in Sweden have examined whether declines in youth alcohol consumption are occurring collectively across the population using school survey data, but have obtained mixed results. Some find evidence of collectivity 3, 4, whereby youth declines in alcohol consumption are proportionate across light‐, moderate‐ and heavy‐drinking 11–15 3, 15–16 18 and 17–18‐year‐olds 4. Further support for collectivity theory comes from a Norwegian study that showed that alcohol consumption increased among all Norwegian 16–17‐year‐old drinkers between 1995 and 2011, in line with population trends 19. Conversely, other studies conclude that they find evidence of polarization and report increases in consumption 20 or no change in consumption among the heaviest drinkers 21, alongside declining consumption for lighter drinkers.

These inconsistent findings have been attributed to differences between the studies’ data sources 20 and analytical approaches 21. Specifically, those studies that find evidence in support of collectivity largely analyse cross‐sectional alcohol consumption data using ordinary least squares (OLS) regression 22, whereas a newer study using a more technical and robust quantile regression model does not find evidence in favour of collectivity 21. Another factor that confuses discussion in this area is uncertainty as to the precise predictions of Skog's theory; in some instances, results that are similar or show only small differences are presented as evidence for both collective consumption trends and their antithesis, polarized consumption trends 3, 21. This confusion occurs because authors differ in their definitions of collectivity, with some considering that the magnitude of change needs to be roughly equivalent throughout different drinking groups, while others require only that trends go in the same direction 23. There has also been confusion concerning what constitutes polarization, with some authors suggesting that stability in trends among the heaviest drinkers alongside declines in the majority amount to polarization 21. To clarify debates around what constitutes collectivity, and in line with a recently published article 24, we distinguish between ‘hard’ and ‘soft’ collectivity. Hard collectivity requires proportional trends at all percentiles of the consumption distribution to be equivalent in magnitude and direction. Soft collectivity requires that trends across percentiles are in the same direction or are stable over time, allowing for a scenario where collective change occurs but declines in alcohol consumption are proportionally smaller or absent among heavier drinkers when compared to lighter drinkers (or vice versa). A third possibility is that we observe polarization in youth drinking trends where there are upward trends at one part of the consumption distribution and downward trends at another. Under this clearer definition of collectivity and polarization, the Swedish evidence is more consistent than the associated research reports suggest, and points towards soft collectivity in the reductions in youth drinking since the early 2000s 3, 4, 21.

To date, examinations of collectivity have primarily occurred in Sweden 3, 4, 18, 20, 21, with one study in Norway 19. Further international work examining whether reductions in youth drinking occur collectively in a broader range of contexts and countries is now required to understand the international public health implications. Therefore, the primary aim of this paper is to test whether the declining trend in youth drinking among 11–15‐year‐olds in England is present and of consistent magnitude throughout the consumption distribution. We also examine whether there are sex and age differences in consumption trends among different percentiles of the consumption distribution, as a recent review demonstrated that declines in youth alcohol consumption are larger for boys than for girls and larger among younger drinkers 25. This may arise from differences in the collectivity of trends throughout the distribution (e.g. the reduction in consumption among heavier‐drinking boys may be larger than for heavier‐drinking girls). Finally, we examine whether declines in consumption trends in all percentiles are in line with overall population declines in consumption as predicted by collectivity theory 16.

## Methods

As described above, we use the following terms to describe our results: (i) hard collectivity—no significant difference in the scale of consumption declines between percentiles; (ii) soft collectivity—declines in all percentiles but significant differences in the magnitude of the decline between percentiles or declines in some percentiles and stability in others; and (iii) polarization—significant differences in the direction of trends with some percentiles increasing consumption and others decreasing consumption.

### Data

The Smoking, Drinking and Drug Use Among Young People Survey (SDD) is a repeat cross‐sectional, school‐based survey in England 6. For the present analyses, SDD data are used from 2001, when the overall decline in alcohol consumption in this survey began. Survey data were collected annually between 2001 and 2016, although there was no survey in 2015 due to funding constraints. In total, this provides 15 waves of data over 16 years, with a combined sample size totalling 124 843.

In each survey year, secondary schools in England are selected to participate using a multi‐stage, stratified sampling method. The data are comparable across years, with few major changes to the sampling, mode of administration or questionnaire over the survey period 26. The majority of secondary schools are eligible to participate in the SDD. Only very small schools, special educational needs (SEN) schools and pupil referral units (special units for students removed from mainstream education, often for behavioural reasons) are excluded.

Between 3000 and 12 000 students, aged from 11 to 15 years, respond to the survey at each wave. Students are randomly selected within schools, such that approximately 30 children from each school participate. In 2016, the sampling method changed slightly and participants were sampled in classes, rather than individual students being randomly sampled from within the school. Three mixed‐ability classes, one from years 7 (aged 11–12) and 8 (aged 12–13) and two from years 9, 10 and 11 (aged 13–15), were randomly selected within each school.

Students self‐completed the survey under examination conditions. Each survey includes a core section of questions focused on pupils’ experiences of smoking, drinking and drug use and retrospective week‐long drinking and smoking diaries. The drinking diary measures the amount of different types of alcoholic drinks (e.g. beer, wine, spirits) consumed in the last 7 days. For example, students are asked to record how many pints, half‐pints, large cans, small cans and bottles of beer, lager or cider they have drunk in the last 7 days. This is then converted into UK units of alcohol (1 unit = 8 g ethanol).

### Measures

The dependent variable was the number of UK units of alcohol consumed during the diary week. Year was entered as a linear variable and values ranged from 2001 to 2016, with no cases for 2015 as there was no survey in this year. Changes were made across all UK national surveys in 2007 to account for shifts in the typical size and strength of alcoholic drinks. As such, the estimates of alcohol units consumed that are reported pre‐ and post‐2007 are not directly comparable and a dummy variable (coded as 0 = pre‐2007 and 1 = post‐2007) was included in the analysis to account for this. We also examine sex (1 = male, 2 = female) and age (11–13‐year‐olds = 1 and 14–16‐year‐olds = 2) differences in consumption trends.

### Analysis

The analysis plan for this study was not pre‐registered, and the findings should be considered exploratory. The data were analysed using simultaneous quantile regression models. Quantile regression estimates the dependent variable at different points on its distribution simultaneously (e.g. at the 50th and 75th quantiles), rather than just at its mean, as in OLS models. Previous studies have predominantly used OLS regression to test for collectivity in consumption trends 3, 4, 20. However, as described by Zeebari *et al*. 21, quantile regression offers distinct advantages over OLS. Quantile regression enables the drinking behaviour of different percentiles of the consumption distribution to be modelled and is more robust than OLS regression, as parametric assumptions of heteroscedasticity and normality, which are commonly violated in alcohol consumption distributions, do not have to be met. As such, quantile regression is appropriate to use with both log‐transformed and untransformed data, which enables the modelling of both the rate of change and the absolute change in mean consumption.

We used quantile regression to estimate year effects (i.e. the slope of the consumption trend) for every fifth percentile (5th–95th). Due to concerns about extreme and potentially unreliable consumption values, we did not look at consumption trends in drinkers in the consumption distribution above the 95th percentile. Mean weekly units consumed was logged to permit examination of relative rather than absolute consumption changes. Analyses using unlogged data are also reported in the Supporting information, Table S1.

Although Skog does not specify whether or not abstainers form part of the consumption distribution, the proportion of respondents who are abstainers matters for this analysis. Increasing rates of abstention contribute significantly to temporal declines in alcohol consumption 23. In the SDD data, rates of abstention increased during the survey period from 73 to 93%. This high and increasing level of abstention creates two problems for our analysis. First, a large proportion of percentiles were at zero units consumed in all years, and therefore analyses of trends at those percentiles would have been uninformative. Secondly, simply excluding all abstainers would have meant not accounting for the variation in the proportion of the sample who were abstainers over time. For example, the 40th percentile in the 2001 distribution was not comparable to the 40th percentile in the 2010 distribution. To provide informative estimates at a larger number of percentiles and to ensure that those percentiles were comparable over time, we sought to exclude a consistent proportion of the sample as abstainers in each year. To do this, we determined the lowest abstention rate across all years, which was 73% in 2001. We then excluded 73% of the population, all of whom are abstainers, in every year. After the 73% of abstainers were removed from each year we were left with a sample of *n* = 38 776. Due to some students not responding to the drinking diary (*n* = 6835), age (*n* = 45) and sex (*n* = 83) questions, the main analysis was conducted on 31 882 full cases. Small random numbers between 0 and 0.99 were added to each of the consumption values of all respondents to allow log‐transformations.

In order to determine whether declines in drinking among the consumption distribution differed by sex and age, we first tested for sex and age differences in the overall population‐level trends with two linear regression models, with sex × year and age × year interaction terms as the independent variable and mean alcohol consumption as the dependent variable. In both instances these interactions were significant, so we included sex × year and age × year interaction terms in the quantile regression models.

Analyses were carried out using the *sqreg* command in Stata version 15. Initial descriptive analyses used weighted data, whereas the quantile models were estimated on unweighted data, as the *sqreg* command cannot incorporate sampling weights. In this instance, sampling weights are unlikely to have a major impact on results, as the SDD uses a robust sampling strategy. The *sqreg* command produces bootstrapped errors; we used 20 bootstraps in the estimation process. In order to examine whether the magnitude of the decline differed significantly between different percentiles of the consumption distribution, post‐estimation Wald tests were conducted using the *test* command.

Finally, in line with collectivity theory 16, we examined whether declines in each percentile were in line with the overall population declines in alcohol consumption. We ran a quantile regression model, with logged overall population mean annual consumption as the independent variable and logged consumption within five percentiles (25th, 50th, 75th, 90th and 95th—those determined by Skog as being light, medium, moderate, near‐heavy and heavy drinkers, respectively) as the dependent variable.

### Sensitivity analyses

A sensitivity analysis reporting a quantile model with unlogged consumption values is reported in the Supporting information. As described in the Measures section, due to changes in the way that alcohol units were calculated in 2007, a pre‐/post‐dummy coded variable was included in the main analysis. However, this change in units coincided with a steepening of the decline in alcohol consumption in 2008. As such, the main analysis was repeated without the dummy variable in order to ensure that the inclusion of the pre‐/post‐variable was not masking an acceleration in the trend.

## Results

**Figure 1:**
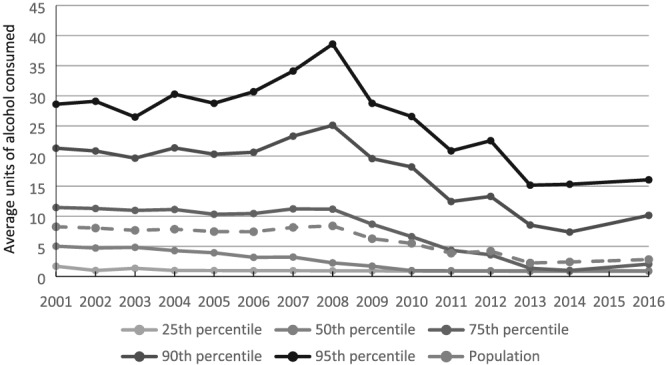
Weighted average units of alcohol consumed by year and percentile with 73% of the population, all of whom were abstainers, excluded. Mean logged consumption in units per year

See Table 1 for mean consumption values, *n*s and response rate for each survey year. Figure 1 shows that the mean number of units consumed by drinkers decreased at the population‐level and throughout the consumption distribution between 2001 and 2016. This is the capped consumption distribution with most abstainers removed, as described above. The overall population average shows that average consumption fell from 8.2 to 2.8 units between 2001 and 2016. Soft collectivity is indicated by the descriptive analyses; average alcohol consumption is declining across all featured percentiles, but the magnitude of this decline seems to be different. Among the lightest drinkers at the 10th percentile, consumption fell from 1.0 units per week in 2001 to 0.8 units per week in 2016. Among the heaviest drinkers at the 95th percentile, consumption fell from 28.6 units per week in 2001 to 16.1 units per week in 2016.

**Table 1:** Descriptive statistics.

Year	*n* a	Overall mean consumption (SD)	Response rate^b^
2001	2396	80.84 (0.22)	61%
2002	2556	80.55 (0.22)	63%
2003	2651	80.28 (0.20)	65%
2004	2479	80.48 (0.23)	62%
2005	2352	80.09 (0.24)	60%
2006	2041	80.05 (0.28)	55%
2007	2019	80.84 (0.31)	53%
2008	1943	90.08 (0.34)	51%
2009	1919	70.18 (0.27)	47%
2010	1864	60.37 (0.27)	41%
2011	1691	40.49 (0.20)	42%
2012	1956	40.89 (0.25)	43%
2013	1293	20.91 (0.16)	38%
2014	1683	30.20 (0.19)	35%
2016	3039	30.81 (0.14)	26%

a *n* refers to capped sample after 73% of students (all abstainers) were excluded from each year.

b Declining overall response rate was due predominantly to declining response rates among schools, individual response rates within schools were similar across years. The main reasons given by schools for not taking part were focused on time, resources and the large number of school surveys being conducted. SD = standard deviation.

A linear regression model with a sex × year interaction term as an independent variable demonstrated that the slope of the population‐level consumption trend differed by sex [β = 0.01, standard error (SE) < 0.01, *P* = 0.001, confidence intervals (CIs) = < 0.01, 0.02] such that the relative change in youth alcohol consumption was larger among males than females during the study period. Similarly, a linear regression model showed that population consumption trends differed by age (β = −0.01, SE < 0.01, *P* < 0.001, CIs = −0.02, −0.01), whereby the relative change in youth alcohol consumption was larger among older drinkers. As such, sex × year and age × year interaction terms were included in the quantile regression analyses in order to determine whether the sex and age differences are seen throughout the distribution or only among lighter/heavier drinkers.

The quantile regression analysis, shown in Table 2, indicated that the average number of weekly units consumed declined significantly across all modelled percentiles of the consumption distribution between 2001 and 2016. The coefficients represent the percentage change in mean consumption each year at the corresponding percentile of the consumption distribution (e.g. consumption at the 5th percentile fell by 1.0% each year). The relative change appears largest in drinkers between the 65th and 95th percentiles of consumption and peaks at the 90th percentile where consumption fell by 21% each year. There were significant sex differences in consumption trends between the 25th and 85th percentiles, where female consumption declined at a slower rate than male consumption. There were no significant sex differences in consumption trends at any other percentiles (Table 2). There were significant age differences in consumption trends at nearly all percentiles (10th–95th), whereby declines in older drinkers were greater than in younger drinkers.

**Table 2:** Results of simultaneous quantile regression with capped abstainers and log‐transformed consumption (*n* = 31 882).

Percentile	Coefficient	SE	*T*	*P* relating to year	CIs	*P* relating to sex × year interaction	*P* relating to age × year interaction
5	−0.01	< 0.001	−53.12	< 0.001^a^	−0.01, −0.01	0.772	0.162
10	−0.01	< 0.001	−46.94	< 0.001	−0.01, −0.01	0.784	< 0.001
15	−0.01	< 0.001	−35.86	< 0.001	−0.01, −0.01	0.628	< 0.001
20	−0.01	0.001	−10.93	< 0.001	−0.01, −0.01	0.361	< 0.001
25	−0.01	0.001	−8.03	< 0.001	−0.01, < −0.01	0.039	< 0.001
30	−0.01	0.001	−9.84	< 0.001	−0.01, < −0.01	0.019	< 0.001
35	−0.01	0.001	−12.11	< 0.001	−0.01, −0.01	0.008	< 0.001
40	−0.01	0.001	−11.79	< 0.001	−0.01, −0.01	0.002	< 0.001
45	−0.01	0.001	−13.47	< 0.001	−0.01, −0.01	< 0.001	< 0.001
50	−0.02	0.001	−15.95	< 0.001	−0.02, −0.01	< 0.001	< 0.001
55	−0.02	0.002	−14.26	< 0.001	−0.02, −0.02	< 0.001	< 0.001
60	−0.03	0.002	−12.90	< 0.001	−0.03, −0.02	< 0.001	< 0.001
65	−0.05	0.004	−12.77	< 0.001	−0.06, −0.04	< 0.001	0.030
70	−0.08	0.005	−15.82	< 0.001	−0.09, −0.07	< 0.001	0.001
75	−0.11	0.004	−26.01	< 0.001	−0.12, −0.11	< 0.001	< 0.001
80	−0.15	0.004	−38.30	< 0.001	−0.16, −0.14	< 0.001	< 0.001
85	−0.19	0.004	−46.60	< 0.001	−0.20, −0.18	< 0.001	< 0.001
90	−0.21	0.017	−12.76	< 0.001	−0.24, −0.18	0.279	< 0.001
95	−0.16	0.012	−12.94	< 0.001	−0.18, −0.13	0.325	< 0.001

The significant declines in consumption amongst the lowest percentiles (some of which will be abstainers) is due to the addition of a random small number before transformation and differing levels of abstention across years. CI = confidence interval; SE = standard error.

### Soft versus hard collectivity

Table 3 shows that the coefficients for all percentiles are negative, and therefore there is no evidence of polarization. As comparing coefficients at all percentiles against each other is impractical, we selected the 50th and 90th percentiles as the points of comparison when assessing whether the trends represent soft or hard collectivity as these percentiles represented, respectively, the point after which the coefficients start to increase and the point of the largest decline.

**Table 3:** *P*‐values for Wald significance tests comparing trends at different percentiles.

Percentiles	50th percentile	90th percentile
5	**< 0.001** a	**< 0.001**
10	**< 0.001**	**< 0.001**
15	**< 0.001**	**< 0.001**
20	**< 0.001**	**< 0.001**
25	**< 0.001**	**< 0.001**
30	**< 0.001**	**< 0.001**
35	**< 0.001**	**< 0.001**
40	**< 0.001**	**< 0.001**
45	**< 0.001**	**< 0.001**
50	–	**< 0.001**
55	**< 0.001**	**< 0.001**
60	**< 0.001**	**< 0.001**
65	**< 0.001**	**< 0.001**
70	**< 0.001**	**< 0.001**
75	**< 0.001**	**< 0.001**
80	**< 0.001**	**< 0.001**
85	**< 0.001**	**< 0.001**
90	**< 0.001**	–
95	**< 0.001**	**< 0.001**

a Bonferroni correction applied to correct for multiple comparisons; bold values are significant.

Table 3 shows the results of the post‐estimation Wald significance tests comparing the magnitude of the decline across different percentiles. The decline in consumption for all percentiles from the 50th onwards is significantly larger than the decline in consumption at the 45th percentile and below and significantly smaller than at the 60th percentile and above. Similarly, the decline in consumption at the 90th percentile was significantly greater than at all other percentiles. This is evidence of soft collectivity; alcohol consumption is declining across all percentiles but the magnitude of the decline differs across the consumption distribution.

### Mean consumption and within percentile consumption

**Figure 2:**
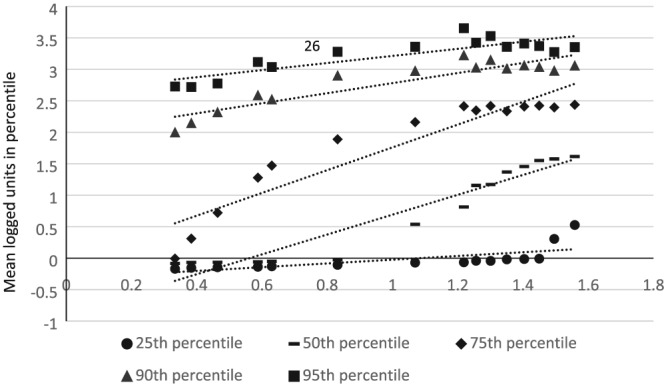
Relationship between overall logged mean consumption and the logged consumption level of selected percentiles

A regression analysis showed that as mean consumption decreased the level of alcohol consumption across all levels of drinking (25th, 50th, 75th, 90th and 95th percentiles) decreased (Table 4). However, in line with soft collectivity, the strength of this relationship was stronger in more moderate drinkers (50th and 75th percentiles); see Fig. 2.

**Table 4:** Quantile regression of overall logged mean consumption and logged consumption within deciles.

Percentile	Coefficient	SE	*T*	*P*
25	0.15	0.002	88.67	< 0.001
50	1.44	0.014	101.81	< 0.001
75	1.55	0.035	44.03	< 0.001
90	0.69	0.023	29.69	< 0.001
95	0.52	0.035	14.59	< 0.001

#### Sensitivity analyses

Please see Supporting information for two sensitivity analyses; capped abstention and unlogged consumption and the main analysis without the pre‐/post‐2007 dummy‐coded control variable.

## Discussion

This paper extends the geographic focus of previous collectivity research and examines whether the decline in youth alcohol consumption, seen in most high‐income countries, is consistent across the consumption distribution in England. Reductions in the average weekly units consumed occurred across the alcohol consumption distribution for 11–15‐year‐olds in England between 2001 and 2016. However, the scale of these reductions differed among percentiles. Proportional reductions in consumption during the study period were significantly largest at the 90th percentile than at lighter or heavier drinking percentiles. This suggests that, although changes in youth alcohol consumption trends in England are collective, they only exhibit soft, rather than hard, collectivity. These findings, taken alongside those from Sweden, which are largely supportive of soft collectivity in youth drinking declines [3,4,41], provide support for making a clear distinction between hard and soft collectivity. Our findings also show some evidence of sex differences in consumption trends; female consumption declined at a slower rate than male consumption overall, and our quantile regression analysis suggests that this is due to differences in trends between the 25th and 85th percentiles of the consumption distribution. Furthermore, there were age differences in consumption trends at nearly all percentiles (10th–95th), whereby declines in consumption were larger in older drinkers. Further analyses of differences in consumption trends across socio‐demographic groups is required to understand the implications of the decline in youth drinking for public health, practice and policy. Finally, declines in consumption in all percentiles were in line with mean decreases in population consumption, although the strength of this relationship differed, providing further evidence of soft collectivity.

This paper provides an important step forward in characterizing the nature of declines in youth drinking in England and extends the geographical focus of previous collectivity research using robust empirical methods and a large nationally representative sample 21. However, it is not without limitations. Unfortunately, school‐level data are not provided in the SDD and data on geographical region was not measured consistently over the time‐frame; as such, it was not possible to control for clustering within schools or geographical region in this analysis. Previous research demonstrates that adolescent non‐responders of surveys are more likely to be heavy consumers than responders 27, potentially because the heaviest drinkers are less inclined to respond to surveys or may not be attending school. We have little understanding of alcohol consumption trends within high‐risk and vulnerable populations, as international research on the decline in youth drinking to date focuses primarily on mainstream samples. There have been reductions in hospitalizations among young people for conditions wholly attributable to alcohol in England 28, but further research examining drinking trends in high‐risk and vulnerable groups remains necessary to establish more robustly whether drinking is also declining in these groups. There are also concerns about the validity of responses from self‐report surveys, as respondents tend to under‐report the amount of alcohol they drink at higher levels of consumption 29 and this may mask evidence of polarization. Further, recent studies show that infrequent drinkers actually underestimate alcohol consumption proportionately more than heavier drinkers 30, 31, which could mask evidence of harder collectivity. Despite a lack of independent verification of self‐reported alcohol consumption data in the SDD, studies which examine adolescents self‐reported drinking generally find the results to be reliable 32, 33, 34. Furthermore, attempts to check the reliability of self‐reported smoking and drug use data through analysing cotinine samples and the inclusion of questions about a fictional drug in the SDD demonstrate that respondents are largely honest 6. There is also no reason to assume that respondents have become more likely to under‐ or overestimate consumption than in previous years, although changing norms around youth alcohol use may affect this. On balance, we judge that it is likely that the reported trends are reflective of real‐world declines in consumption.

Our results provide no evidence of emerging polarization in youth alcohol consumption trends, and this could have important implications both in terms of public health and policy recommendations. Alcohol can cause a series of harmful effects, particularly on adolescents, and has been linked to poorer cognitive development 9 alcohol use disorders later in life 9, 10, accidents 11 and risky sexual behaviour 11. As such, declining youth drinking throughout all levels of consumption could carry both short‐ and long‐term population health benefits. Furthermore, we find the largest decline in consumption among heavy drinkers in the 90th percentile, which suggests that the positive benefits of declines in alcohol consumption may be maximized. These findings could also have implications in determining how policies should target alcohol‐related harms in young people, and suggest that targeted campaigns at heavier‐drinking youths may not be necessary. Unless further evidence suggests declines in consumption are small or non‐existent among vulnerable young people outside mainstream school samples, public health strategies should continue to aim to reduce youth drinking across the population. A review of international evidence suggests that the most effective measures to continue to promote declines in youth drinking are policies which restrict the availability and marketing of alcohol 35.

These findings are limited to 11–15‐year‐olds, and it is possible that polarization may still be observed after the age of 15. This is particularly the case after age 18, as university attendance has been linked with greater alcohol consumption and the mechanisms driving this may interact with those driving the downward trend in youth drinking 36, 37. It is as yet unclear whether there have been increases in consumption in different groups of young people over time. Rather, it could be that, although university students may occupy the top percentiles of the consumption distribution, they may be drinking less than comparable students in previous years. Further research examining how declines in alcohol consumption vary throughout the consumption distribution among young people aged 18–24 would therefore be of value.

## Conclusion

Declines in youth alcohol consumption occur collectively among 11–15‐year‐olds in England, although the magnitude of the decline in consumption differs significantly between percentiles of the consumption distribution. The proportional declines are largest in heavier drinkers and, as such, the potential public health benefits of declining youth drinking may be recognized. These results also support the need for a more nuanced definition of collectivity, with more meaningful conceptual categories of hard and soft collectivity.

## Declaration of interests

None.

## Supporting information


**Table S1** Results of simultaneous quantile regression with capped abstainers and untransformed consumption.
**Table S2** Results of simultaneous quantile regression with capped abstainers and logged consumption but without the dummy pre/post‐2007 variable.
