# Supplementary material for: The decline in youth drinking in England—is everyone drinking less? A quantile regression analysis
Source: Addiction. 2019 Dec 1;115(2):230–8. doi: 10.1111/add.14824 (PMC7004203; doi:10.1111/add.14824)
Supplement: Supplementary file 1 — Table S1 Results of simultaneous quantile regression with capped abstainers and untransformed consumption. Table S2 Results of simultaneous quantile regression with capped abstainers and logged consumption but without the dummy pre/post‐2007 variable. [file ADD-115-230-s001.docx]

**Supplementary Materials**

For the model with capped abstention and unlogged consumption (Supplementary Table 1) the pattern differed slightly to the main analysis. There were significant declines in consumption at all percentiles, this started to increase from the 60^th^ percentile (β = -.04, SE = .01, CI’s = -.05, -.03) and was highest in the 95^th^ percentile (β = -1.95, SE = .10, CI’s = -2.14, -1.75). This is indicative of a decline in consumption at the 60^th^ percentile and 95^th^ percentile equivalent to a decrease of .04 and 1.89 units a week respectively. The greater drop in absolute consumption amongst the heaviest drinkers is reflective of the different baseline consumption levels between higher and lower percentiles, which provide more or less scope for change.

The sensitivity analysis without the pre/post 2007 dummy variable (Supplementary Table 2) showed a similar pattern of results as the main analysis. The coefficients started increasing from the 60^th^ percentile (β = -.04, SE = .003, CI’s = -.04, -.03) and the largest change was at the 85^th^ percentile (β = -.18, SE = .002, CI’s = -.18, -.17).

We did not test for significant differences between the scale of the decline at different percentiles of the consumption distribution in the sensitivity analyses.

Supplementary Table 1: *Results of simultaneous quantile regression with capped abstainers and untransformed consumption.*

| Percentile | Coefficient | SE | *T* | *P –* relating to year | CI’s | *p* - relating to Sex*Year Interaction ^1^ | *p* - relating to Age*Year Interaction |
| --- | --- | --- | --- | --- | --- | --- | --- |
| 5 | -.01 | <.001 | -58.23 | <.001 | -.01, -.01 | .932 | .033 |
| 10 | -.01 | <.001 | -41.67 | <.001 | -.01, -.01 | .606 | <.001 |
| 15 | -.01 | <.001 | -39.43 | <.001 | -.01, -.01 | .767 | <.001 |
| 20 | <-.01 | <.001 | -8.99 | <.001 | -.01, <-.01 | .452 | <.001 |
| 25 | <-.01 | .001 | -6.69 | <.001 | -.01, <-.01 | .051 | <.001 |
| 30 | <-.01 | .001 | -7.64 | <.001 | -.01, <-.01 | .069 | <.001 |
| 35 | -.01 | .001 | -8.70 | <.001 | -.01, <-.01 | .047 | <.001 |
| 40 | -.01 | .001 | -10.89 | <.001 | -.01, -.01 | .030 | <.001 |
| 45 | -.01 | .001 | -12.35 | <.001 | -.01, -.01 | .007 | <.001 |
| 50 | -.01 | .001 | -12.21 | <.001 | -.02, -.01 | .001 | <.001 |
| 55 | -.02 | .004 | -6.73 | <.001 | -.03, -.02 | <.001 | <.001 |
| 60 | -.04 | .007 | -5.49 | <.001 | -.05, -.03 | <.001 | <.001 |
| 65 | -.06 | .022 | -8.62 | <.001 | -.08, -.05 | <.001 | <.001 |
| 70 | -.10 | .011 | -9.09 | <.001 | -.12, -.08 | <.001 | <.001 |
| 75 | -.21 | .014 | -14.39 | <.001 | -.24, -.18 | <.001 | <.001 |
| 80 | -.40 | .015 | -25.77 | <.001 | -.43, -.37 | <.001 | <.001 |
| 85 | -.69 | .032 | -21.47 | <.001 | -.75, -.62 | <.001 | .327 |
| 90 | -1.17 | .052 | -22.37 | <.001 | -1.27, -1.06 | <.001 | .069 |
| 95 | -1.95 | .101 | -19.33 | <.001 | -2.14, -1.75 | <.001 | <.001 |

Supplementary Table 2: *Results of simultaneous quantile regression with capped abstainers and logged consumption but without the dummy pre/post 2007 variable.*

| Percentile | Coefficient | SE | *T* | *P –* relating to year | CI’s | *p* - relating to Sex*Year Interaction ^1^ | *p* - relating to Age*Year Interaction |
| --- | --- | --- | --- | --- | --- | --- | --- |
| 5 | -.01 | <.001 | -79.42 | <.001 | -.01, -.01 | .914 | .371 |
| 10 | -.01 | <.001 | -61.51 | <.001 | -.01, -.01 | .952 | <.001 |
| 15 | -.01 | <.001 | -62.01 | <.001 | -.01, -.01 | .805 | <.001 |
| 20 | -.01 | <.001 | -58.46 | <.001 | -.01, -.01 | .215 | <.001 |
| 25 | -.01 | <.001 | -58.07 | <.001 | -.01, -.01 | .096 | <.001 |
| 30 | -.01 | <.001 | -51.16 | <.001 | -.01, -.01 | .043 | <.001 |
| 35 | -.01 | <.001 | -48.35 | <.001 | -.01, -.01 | .001 | <.001 |
| 40 | -.01 | <.001 | -30.99 | <.001 | -.01, -.01 | .001 | <.001 |
| 45 | -.01 | .001 | -23.39 | <.001 | -.01, -.01 | <.001 | <.001 |
| 50 | -.01 | .001 | -13.27 | <.001 | -.02, -.01 | <.001 | <.001 |
| 55 | -.02 | .002 | -14.06 | <.001 | -.02, -.02 | <.001 | <.001 |
| 60 | -.04 | .003 | -11.79 | <.001 | -.04, -.03 | <.001 | <.001 |
| 65 | -.07 | .004 | -18.68 | <.001 | -.07, -.06 | <.001 | .002 |
| 70 | -.09 | .004 | -23.33 | <.001 | -.10, -.08 | <.001 | <.001 |
| 75 | -.12 | .003 | -35.34 | <.001 | -.12, -.11 | <.001 | <.001 |
| 80 | -.15 | .003 | -45.15 | <.001 | -.15, -.14 | <.001 | <.001 |
| 85 | -.18 | .002 | -70.19 | <.001 | -.18, -.17 | <.001 | <.001 |
| 90 | -.17 | .010 | -16.01 | <.001 | -.19, -.15 | .281 | <.001 |
| 95 | -.12 | .009 | -12.53 | <.001 | -.13, -.10 | .308 | <.001 |
